# Antineoplastic and Antitrypanosomal Properties of Propolis from *Tetragonula biroi* Friese

**DOI:** 10.3390/molecules27217463

**Published:** 2022-11-02

**Authors:** Samyah Alanazi

**Affiliations:** Department of Clinical Laboratory Sciences, College of Applied Medical Sciences, King Saud University, Riyadh 11433, Saudi Arabia; saalanazi@ksu.edu.sa

**Keywords:** Philippine propolis, antitrypanosomal, anticancer, LC-MS, NMR

## Abstract

Propolis, popularly known as bee glue, is a resinous, sticky substance produced by different bee species across the globe. Studies on the biological properties of propolis from the Philippines are rare. Hence, the current study aims at the chemical characterization of propolis produced by the stingless bees *Tetragonula biroi* Friese from the Philippines and to investigate its antitrypanosomal and anticancer properties. The determination of the chemical composition and characterization of propolis samples was achieved using liquid chromatography–mass spectrometry (LC-MS), -high-performance liquid chromatography–evaporative light scattering detector (HPLC-ELSD), and nuclear magnetic resonance (NMR) spectroscopy. Three major triterpenes were isolated and identified using HRESI-MS and ^1^H/^13^C NMR techniques. The spectral studies confirmed the presence of compounds such as isomangiferolic acid, 27-hydoxymangiferonic acid, and 27-hydroxyisomangiferolic acid. All crude propolis samples, isolated fractions, and pure compounds demonstrated moderate antitrypanosomal and anticancer properties compared to control drugs. Amongst the tested compounds, 27-hydoxymangiferonic acid exhibited the highest antitrypanosomal activity at a concentration of 11.6 µg/mL. The highest anticancer effect was demonstrated by the Ph-2 fraction, followed by 27-hydroxyisomangiferolic acid, with IC50 values of 129.6 and 153.3 µg/mL. Thus, it can be concluded that the observed biological activity of Philippine propolis is due to the combinatorial effect or synergistic action of the active compounds 27-hydoxymangiferonic acid and 27-hydroxyisomangiferolic acid.

## 1. Introduction

The onset and evolution of infectious and chronic diseases has made the need for the discovery and development of novel drugs paramount in disease therapy. Inevitably, natural products are the first choice for discovery of novel drugs, as they are enriched with bioactive ingredients with pharmacological properties [1,2,3]. Propolis is an apicultural product that is used as an alternative medicine for disease control in different parts of the world. Globally, propolis has garnered the interest of the scientific community due to its pharmacological properties. It is a resinous material collected by bees from various plant exudates [4]. This bee glue is produced by honeybees in the process of sterilizing the hive environment [5] and ensures a healthy hive environment for the bee colony due to its antibiotic properties. Interestingly, bees use propolis to achieve strong architecture in a bee hive by sealing cracks and narrowing the entrances so as to provide protection from intruders. Propolis is also used in the embalming the dead organisms. The applicability of propolis in medical sciences is not a new adventure. It has a long history; by 300 B.C, propolis had been used as a folk medicine to combat microbial infections [3]. Nonetheless, its applicability is now widespread, ranging from pharmaceuticals and cosmetics to the food and beverage industries [6].

Propolis is produced by varied bee species, including Apis mellifera and stingless bees (Meliponini). There are approximately 500 species within the stingless bee genus [7,8,9]. *Melipona* and the *Tetragonula* are the two genera of stingless bees. The *Tetragonula biroi* Friese stingless bee species is one of the known species of honeybees that are endemically living in the Philippines. *Tetragonula* is the genus name, and its complete name is *Tetragonula biroi* Friese. It is abundantly found in the Bicol region in the Philippines. Propolis from stingless bees is well-known for its antimicrobial, antitumor, and antioxidant activities [10,11]. Cancer is the world’s second major cause of death and a serious global health issue. The use of chemotherapeutic drugs reduces the cancer rate but increases the risk of infection. Hence, natural compounds with therapeutical potential are in demand. Globally, several reports on stingless bee propolis have shown cytotoxicity against cancerous cell lines. The propolis of *Scapto depilis* and *Melipona quadrifasciata* from Brazil demonstrated cytotoxicity against erythroleukemic cells [12], and stingless bee propolis extract of *Tetragonula minor* from Vietnam exhibited cytotoxicity against PANC-1 human pancreatic cancer cells [13]. The anticancer potential of Chinese propolis studied on human breast cancer cells exhibited significantly increased ROS, NF-*κ*B, and p65 expressions, which are prerequisites for cancer development [14]. The antibacterial and anticancer effects of propolis from different countries have been documented previously, yet investigations on the antiparasitic and anticancer/antineoplastic properties of Philippine propolis are scarce. The bioactivity of propolis varies with geographical origin and climatic conditions. Hence, the present study was undertaken to characterize and evaluate the bioactivity of propolis from the stingless bees *Tetragonula biroi* Friese obtained from the Philippines. The outcomes of this study increase our knowledge of Philippine propolis as a potential nutraceutical agent with therapeutic benefits against human leukemia and parasitic infections.

## 2. Results

### 2.1. Chemical Profiling of Crude Philippine Propolis

**Figure 1:**
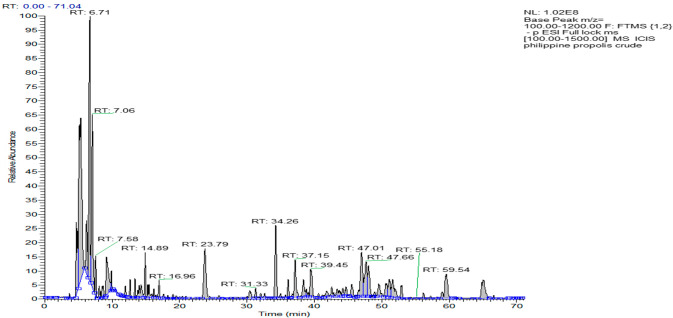
Chromatogram of the ethanolic extract of Philippine propolis on LC-MS.

**Figure 2:**
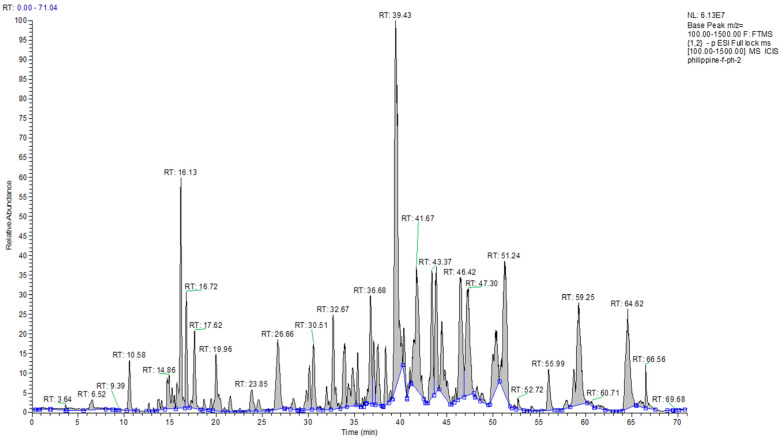
Chromatogram view of the Philippine fraction (Ph-2) on LC-MS.

The results from the HPLC-UV-ELSD detection revealed the presence of compounds lacking chromophores, such as terpenoids, fats, or other compounds, in the content of the crude PP sample. Compounds that absorbed UV were also identified but at lower intensities (Figure 1). An overall picture of the crude sample constituents was obtained through the ^1^H NMR spectra, which indicated strong responses for aliphatic protons. Alkene protons were important, and the main signals in the spectra were indicative of the presence of terpenoids. The NMR spectra, in addition to major signals due to terpenoids, revealed the presence of phenolic compounds but at lower intensities. Figure 1 depicts the LC-MS profiling of the crude PP extract, which had considerable complexity and multiple peaks of varying intensities. Like NMR, the LC-MS analysis also revealed the occurrence of terpenoids as the main constituents of the ethanolic extract. To obtain pure fractions, the ethanolic extract of PP (around 5.3 g) was initially subjected to CC, and elution was sequentially performed based on a gradient profile. The total number of generated fractions was 28. The performance of LC-MS and HPLC-UV-ELSD permitted the identification of the different components, which allowed the combination of fractions. The final number of fractions was ten. The LC-MS and HPLC-UV-ELSD analysis highlighted the presence of the richest constituents in the 877 mg of the Philippine fraction (Ph-2), as indicated in Table 1 and Figure 2. Based on the preliminary data, the compounds were most likely terpenoids. Further, the 877 mg of fraction Ph-2 was subjected to SEC, yielding 61 subfractions (Ph-2-1 to Ph-2-61), which led to the acquisition of three pure compounds (Ph-2-11, Ph-2-14, and Ph-2-20).

### 2.2. Characterization of Ph-2-14 as 27-Hydroxymangiferonic Acid

**Figure 3:**
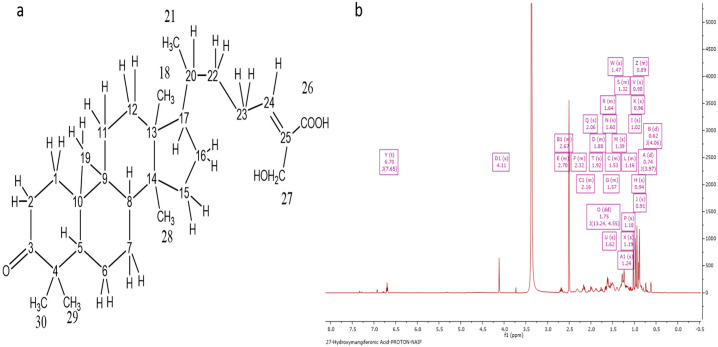
Characterization of Ph-2-14 as 27-hydroxymangiferonic acid. (**a**) Structure of 27-hydroxymangiferonic acid; (**b**) ^1^H NMR spectrum (400 MHz) of 27-hydroxymangiferonic acid (Ph-2-14) in DMSO-*d*_6_.

Following several chromatographic methods, including CC and SEC, Ph-2-14 was obtained from the ethanolic extract of Philippine propolis with a white solid form in appearance. Spraying with a *p*-anisaldehyde-sulfuric acid reagent and subsequently heating caused it to manifest as a single spot on TLC. Elution with the mobile phase of 50% HE in EtOAc gave an Rf of 0.68 on SiGel. The molecular formula C_30_H_45_O_4_ was established based on the fact that the molecular ion [M-H]^−^ was indicated by the negative-mode HRESI-MS spectrum at *m*/*z* 469.33 (Figure 3a).

The ^1^H NMR spectrum (400 MHz) of 27-hydroxymangiferonic acid (Ph-2-14) in DMSO-d_6_ is illustrated in Figure 3b. The proton spectrum of the compound showed an olefinic triplet at δ_H_ 6.70 ppm, two highly shielded cyclopropane protons at 0.62 and 0.74 ppm, a methyl doublet at 1.02, a proton quartet at 1.47 ppm, and an oxygenated methylene group at 4.11 ppm. Other proton signals were typical of a triterpene moiety. The carbon spectrum showed 30 carbon signals, including a saturated cyclic ketone at δ_C_ 215.36 ppm, a carboxylic acid carbon at 168.83, two olefinic carbons at 145.48 (CH) and 132.85 (C), one oxygen-bearing carbon at 55.23 ppm, and five methyl (CH_3_) signals. The protons directly bonded to carbons were obtained from the HSQC spectrum, while the COSY spectrum was used to identify any neighboring proton connectivity.

Finally, the long-range (^3^*J* and ^2^*J*) and one-bond (^1^*J*) proton–carbon correlations were used to confirm the structure and assign the chemical shifts for the compound. Correlations from the oxymethylene protons at C-27 to the carboxylic acid at C-26 indicated they were germinal, while other correlations from H-24 confirmed C-26, C-27, and C-22, and correlations from H-28 and H-29 identified C-3, C-4, C-5, and the germinal methyl carbons at C-4.

### 2.3. Characterization of Ph-2-11 as 27-Hydroxyisomangiferolic Acid

**Figure 4:**
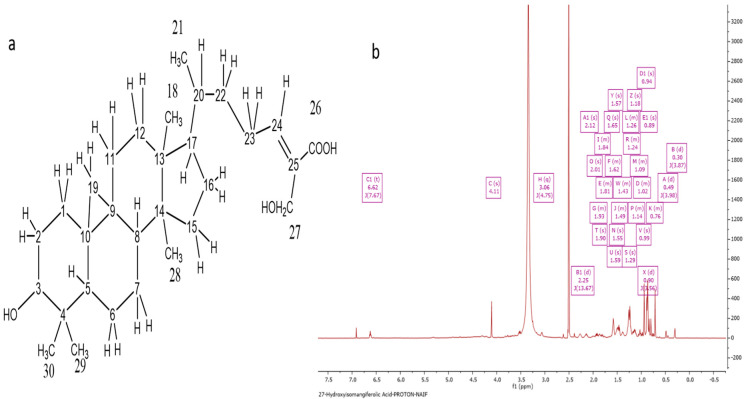
Characterization of Ph-2-11 as 27-hydroxyisomangiferolic acid. (**a**) Structure of 27-hydroxyisomangiferolic acid; (**b**) ^1^H NMR spectrum (400 MHz) of 27-hydroxyisomangiferolic acid (Ph-2-11) in DMSO-d_6_.

The acquisition of Ph-2-11 was accomplished by running a PP ethanolic extract on CC and SEC. A molecular ion, [M-H]^−^, was indicated by the negative mode at *m/z* 471.35 in the HRESI-MS spectrum with a molecular formula of C_30_H_47_O_4_ and an optical rotation value of +23.5° (Figure 4a). The ^1^H NMR spectrum (400 MHz) of Ph-2-11 (Figure 4b) confirmed the presence of 27-hydroxyisomangiferolic acid. Its proton spectrum was identical to that of Ph-2-14, except for the presence of a proton doublet of doublets at δ_H_ 3.06 ppm. The carbon spectrum was also identical, except for the absence of the ketone carbonyl at δ_C_ 215.4 and the presence of an oxymethine carbon at 77.09 ppm. This supports the replacement of the ketone at C-3 with an −OH group.

### 2.4. Characterization of Ph-2-20 as Isomangiferolic Acid

**Figure 5:**
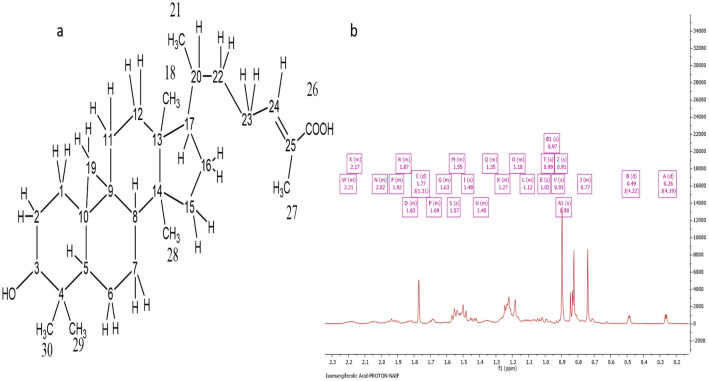
Characterization of Ph-2-20 as isomangiferolic acid. (**a**) Structure of isomangiferolic acid; (**b**) ^1^H NMR spectrum (400 MHz) of isomangiferolic acid (Ph-2-20) in CDCl_3_.

The NMR and HRESI-MS spectral data characterized Ph-2-20 as isomangiferolic acid, with a molecular formula of C_30_H_47_O_3_, derived from the fact that the molecular ion [M-H]^−^ was indicated by the negative-mode HRESI-MS spectrum at *m*/*z* 455.35 with an optical rotation value of +32°. Figure 5a depicts the HRESI-MS spectrum of Ph-2-20. The presence of isomangiferolic acid was confirmed by the ^1^H NMR spectrum (400 MHz) of Ph-2-20 (Figure 5b). The compound’s proton spectrum was identical to that of compound Ph-2-11, except for the absence of the oxymethylene singlet at 4.11 ppm, which was replaced by a methyl singlet at 1.77 ppm (d, *J* = 1.31, 3H). All other proton signals were identical. The carbon spectrum also showed the absence of the oxymethylene carbon at 55.83 and the presence of another methyl carbon signal at 11.99 ppm. By comparing the chemical shifts with literature reports [15], the compound was identified as isomangiferolic acid.

### 2.5. Antineoplastic Properties of Philippine Propolis

The cytotoxicity values of all tested compounds of the PP sample are shown in Table 2. Among the tested compounds, 27-hydroxyisomangiferolic acid and the Ph-2 fraction demonstrated higher cytotoxicity, with IC50 values of 153.3 and 129.6 µg/mL, respectively. It is noteworthy that all tested samples exhibited cytotoxicity at IC50 values > 100 µg/mL in comparison to Pentamidine and Diminazene.

### 2.6. Antitrypanosomal Activity of the Philippine Propolis Sample Tested against T. brucei S427 Strain

Table 3 summarizes the drug sensitivity assay of the PP sample and its fractions on *T. brucei* S427 WT. Crude, fraction, and pure compounds (isomangiferolic acid, 27-hydoxymangiferonic acid, and 27-hydroxyisomangiferolic acid) extracted from the PP sample were tested against *T. brucei*. Pentamidine and Diminazine were used as drug controls. The results showed varying activities against *T. brucei* in the tested samples. Interestingly, 27-hydoxymangiferonic acid as well as the Ph-2 fraction had higher activities, 11.4 and 11.6 µg/mL, respectively, compared to the crude PP sample (22.0 µg/mL). The other pure compounds, isomangiferolic acid and 27-hydroxyisomangiferolic acid, had slightly lower activities in comparison to 27-hydoxymangiferonic acid, with MICs of 21.4 µg/mL and 13.9 µg/mL, respectively. Overall, the crude extract, fractions, and pure compounds had moderate activities against *T. brucei* S427 WT.

## 3. Discussion

The occurrence of terpenoids as the major chemical constituents in Philippine propolis was demonstrated by GC-MS profiling. Data from the ^1^H/^13^C NMR spectrum and spectral studies confirmed the presence of compounds such as isomangiferolic acid, 27-hydoxymangiferonic acid, and 27-hydroxyisomangiferolic acid. Upon testing for antitrypanosomal and anticancer properties, all crude extracts and fractions as well as isolated compounds, viz. isomangiferolic acid, 27-hydoxymangiferonic acid, and 27-hydroxyisomangiferolic acid, exhibited moderate antitrypanosomal and anticancer properties. Amongst the tested compounds, 27-hydoxymangiferonic acid exhibited the highest antiparasitic or antitrypanosomal activity. The highest anticancer effect was demonstrated by the Ph-2 fraction, followed by 27-hydroxyisomangiferolic acid, reflecting that the cytotoxic activity was due to the combinatorial effect of these isolated compounds.

It is evident in the literature that propolis varies in chemical composition, even in the same geographical location. Besides the chemical complexity, propolis can vary in its biological properties, which significantly effects propolis quality. Propolis has been reported to contain over 300 chemical compounds [15,16], but not all compounds have biological activity. Therefore, robust approaches comprising different chromatography and spectroscopic techniques, such as gas chromatography (GC), high-performance liquid chromatography (HPLC), mass spectroscopy (MS), and nuclear magnetic resonance spectroscopy (NMR), are warranted for the standardizing and isolation of pure propolis components and testing its efficacy using clinical trials.

Around the globe, researchers have been involved in investigating the pharmacological activity of propolis from different countries. In the current study, we focused on evaluating the therapeutic potential of Philippine propolis and tested its anticancer and antitrypanosomal properties. The therapeutic potential of propolis from indigenous stingless bees from the Philippines is limited. Few studies had investigated its antimicrobial properties [17,18], whilst more recently, an anticancer property against gastric cancer was also reported [19]. However, little is known about the biological activities of Philippine propolis.

Based on literature data, it was found that the chemical profile varies in different types of propolis from different regions around the world. In the current study, the chemical characterization of propolis resulted in the detection of terpenoids as the major constituents in the propolis sample. The spectral characteristics and the occurrence of terpenoids in the propolis extract obtained here were similar to previous investigations on propolis from Indonesia and the United Kingdom [20,21]. Terpenoids role as antioxidants and promising tools in the treatment of CNS disorders such as Parkinson’s disease has been documented [22]. Based on the spectral data obtained in the current study and evidence from previous reports, three major triterpenes were identified in the ethanolic extract of PP: 27-hydroxymangiferonic acid, 27-hydroxyisomangiferolic acid, and isomangiferolic acid. The full chemical shift assignments observed in the NMR spectra of 27-hydroxymangiferonic acid (Figure 3b) were similar to previous reports [16]. Similarly, in 2017, Nguyen et al. confirmed chemical shifts similar to those documented for 27-hydoxyisomangiferonic acid [13], shown in Figure 4b. Similar bioactive compounds were obtained in a previous report of a study characterizing propolis from *H. fibriata* [21].

It is well-known that cancer is a devastating disease. Chemotherapy is one the major therapeutic approaches for the treatment of benign and metastasized cancer. Together with the increasing death rate and the undesirable side effects with chemotherapy, it is imperative to develop naturally occurring anticancer agents that are safe nontoxic, and have long-term benefits to humans [23]. Propolis extracts are known to inhibit cell growth and the differentiation of tumor cells [24,25]. Further, the cytotoxicity varies in different propolis samples. All pure components exhibited cytotoxicity at IC50 values > 100 µg/mL. The data obtained in the current report are consistent with a 2013 report on Indian propolis by Choudhari et al., where an ethanolic extract of propolis was reported to have cytotoxicity at a concentration of 250 µg/mL in various cancerous cell lines [2]. The observed anticancer activity was in accordance with the ethanolic extract of Brazilian red propolis, which exhibited a strong cytotoxic effect on breast cancer cells (MCF-7) [26]. A study on Turkish propolis by Vatansever et al. in 2009 reported effective antitumor activity at a lower concentration (0.125 mg/mL) [27], and propolis from Saudi Arabia [28] exhibited cytotoxicity at an IC50 of 87.7 µg/mL, thus reflecting the disparity in the levels of cytotoxicity with propolis from different geographical locations. The anticancer property of propolis observed in PP extracts may be attributed primarily to the presence of two major active compounds: 27-hydroxymangiferolic acid and 27-hydroxyisomangiferolic acid. The exact mechanism as to how propolis inhibits the growth of cancerous cells is still unclear. However, cytotoxicity against cancerous cells is assumed to involve one or more of the underlying mechanisms: (a) a flavonoid found in propolis can repress angiogenesis and induce apoptosis, thereby acting against the tumor, or (b) it inhibits cancer progression by targeting multiple signaling pathways, including phosphoinositide 3-kinases (PI3K)/Akt and mitogen-activated protein kinase (MAPK) signaling molecules [29]. Additionally, it is assumed that propolis extracts exhibit cytotoxicity by targeting cell cycle regulators such as cyclin D, cyclin-dependent kinases Cdk-2/4/6, and cyclin-dependent kinase inhibitors, thereby arresting the progression of the cancer cell cycle at the G2/M and G0/G1 phases [30,31]. The anticancer properties exhibited by the isolated compounds 27-hydroxymangiferolic acid and 27-hydroxyisomangiferolic acid are in line with a previous report [21].

Infection from the protozoan parasite *Trypanosoma* is another threat to mankind and animals. The subspecies of *T. brucei* causes sleeping sickness or Human African trypanosomiasis in humans [32], and *T. brucei* and *T. congolense* cause a severe disease in animals, called nagana, or African Animal trypanosomiasis [33]. Bees produce propolis to protect their hives against protozoal infections, as reported by Alotaibi et al. in 2019 [34]. As an example, the current study evaluated the antitrypanosomal property of Philippine propolis. Studies on the antitrypanosomal activity of propolis are scarce. However, propolis from Brazil and Saudi Arabia has been reported to possess strong antitrypanosomal properties. In line with previous evidence, the antitrypanosomal activity of the PP studied in the current report yielded satisfactory results. The observed antitrypanosomal activity observed in the current study was in line with previous findings [20,25,34]. Among the tested compounds, the Ph-2 fraction and 27-hydroxymangiferolic acid had the highest antitrypanosomal activities, at IC50 values of 11.6 and 11.4 µg/mL, respectively, followed by 27-hydroxyisomangiferolic acid, with an IC50 of 13.9 µg/mL. Based on the obtained results, it was confirmed that the activity of PP could be attributed to the presence of two major active compounds: 27-hydroxymangiferolic acid and 27-hydroxyisomangiferolic acid. There are numerous reasons for the decreased activities of the isolated compounds compared to the crude fractions; the retention of the active compound in the column, the distribution of most active constituents over different fractions, or the synergistic interaction between several compounds may be the source of the decreased extract effects compared to the pure fractions.

## 4. Materials and Methods

### 4.1. Chemicals and Reagents

Acetonitrile, methanol, ethyl acetate, water, formic acid (LC-MS-grade), Davisil grade 633 amorphous precipitated silica (pore size 60 A, mesh size 200–425 μm), Sephadex LH-20, p-Anisalde hyde, vanillin, sulfuric acid, and the deuterated solvents chloroform-d (CDCl3) and dimethyl sulfoxide-d6 (DMSO-*d*_6_) were obtained from Sigma-Aldrich (Gillingham, UK). Glass columns for column chromatography was purchased from Rotaflo, Fisher Sceintific, Loughborough, UK. TLC-grade silica gel (60H) and TLC silica gel 60 F254 precoated aluminum sheet and the HPLC-grade solvents ethyl acetate, methanol, acetonitrile, n-hexane, and absolute ethanol were ordered from Merck, Darmstadt, Germany. Alamar blue^®^ BUF 012B (AbD Serotec^®^, Oxford, UK), HMI-9 medium (Invitrogen, Oxford, UK), RPMI-1640 (Lonza, Verviers, Belgium), L-glutamine (Life Tech, Paisley, UK), Penicillin/Streptomycin (Life Tech, Paisley, UK), fetal bovine serum (FBS) (Sigma-Aldrich, Gillingham, UK), U937 cell cultures (obtained from ECACC, Porton Down, Salisbury, UK), 96-well plates (Corning^®^, Sigma-Aldrich), and a plate reader (Perkin Elmer, Buckinghamshire, UK) were also obtained.

### 4.2. Propolis Collection and Preparation for LC-MS

The propolis from the Philippine stingless bee *Tetragonula biroi* Friese was collected from a market near the Honey Bee Center, Laguna, the Philippines (14°3′46″ N, 121°14′53″ E). Raw propolis was collected in an airtight container and kept at −18 °C for 24 h. Later, propolis chunks were shipped to Saudi Arabia in ice-cold conditions. Prior to extraction techniques, propolis was freed of impurities, such as pollen, wood, the dead remains of bees, etc., and fragmented using a mortar and pestle.

### 4.3. Extraction and Purification of Propolis

#### 4.3.1. Extraction

Approximately 30 g of the propolis sample was extracted three times under sonication with 150 mL of ethanol at room temperature for 60 min. The extracts were combined, the solvent was evaporated using a rotary evaporator, and the residue was weighed.

#### 4.3.2. Purification

For the isolation and purification of the propolis sample, the crude extract was subjected to different chromatographic methods, including column chromatography (CC) and size-exclusion chromatography (SEC) [35,36].

##### Column Chromatography

For CC, silica gel 60 with a mesh size of 200–425 μm was used. The wet packing method was adopted for packing the column with approximately 50 g of silica slurry, and the solvent with the lowest polarity (i.e., hexane) was mixed before pouring and packing in a suitably sized glass column, e.g., (55 × 3 cm). Air bubbles were eliminated by tapping the column. Excess solvent was permitted to pass through, and the column was allowed to settle down. Approximately 5 mL of ethyl acetate was used to dissolve about 3 g of propolis extract, which was then mixed with coarse silica (6 g) and dried under a vacuum hood. This was followed by the loading of the dried extract on the column top. This was followed by the sequential performance of elution using 200 mL of hexane, ethyl acetate, and methanol mixtures as follows: hexane/ethyl acetate (80:20), hexane/ethyl acetate (60:40), hexane/ethyl acetate (40:60), hexane/ethyl acetate (20:80), ethyl acetate and then ethyl acetate/methanol (80:20), ethyl acetate/methanol (60:40), ethyl acetate/methanol (40:60), and ethyl acetate/methanol (20:80). A rotatory evaporator was used to collect and concentrate the fractions, which were then aggregated via an HPLC-UV-ELSD analysis based on similar chemical profiles. HPLC-UV-ELSD was performed on an Agilent 1100 system (Agilent Technologies, Waldbronn, Germany) using a reverse-phase C18 column with water and acetonitrile as the mobile phase. The interpretation of the data was carried out using Clarity software (Data Apex).

##### Size-Exclusion Chromatography

The further purification of fractions from CC was carried out using SEC. The preparation of a slurry of Sephadex LH 20 involved the overnight suspension of the stationary phase in 50:50 dichloromethane/methanol for nonpolar fractions and in 100% methanol for polar fractions. This slurry was then used to pack a 2 × 100 cm glass column covered with cotton wool. After the settling of the packed bed, the solvent level exceeded the top of the bed, and a Pasteur pipette was used for the cautious application of the sample intended for fractionation following dissolution in the minimal quantity possible of the solvent employed for column packing. Elution was performed and completed with 100% MeOH in an isocratic manner, and small vials of around 1 mL were used for the collection of the fractions.

### 4.4. Structure Elucidation

#### 4.4.1. Nuclear Magnetic Resonance

To determine the structure of a compound, the method that is usually adopted is nuclear magnetic resonance (NMR). To identify and determine the compounds present in the fractions, one- and two-dimensional experiments were carried out. The acquisition of the NMR data was made possible by a JEOL (JNM LA400) spectrometer (400 MHz) at SIPBS and a Bruker Avance 300 (400 MHz) spectrometer with tetramethylsilane (TMS) as the internal standard. The compounds were prepared in deuterated solvents, such as CDCl3 and DMSO-d6, based on their solubilities. Then, 500–600 μL of a suitable solvent was used for the dissolution of 10 mg of every sample, which were then poured into typical NMR tubes (5 × 178 mm) to a depth of around 4 cm. The NMR spectral data were obtained using MestReNova software 8.1.2 (Mestrelab Research, A Coruña, Spain), and ChemBioDraw Ultra, Version 14 (PerkinElmer, Yokohama, Japan), was employed for illustrating figures of the structures of the isolated compounds.

#### 4.4.2. LC-MS

Similar to NMR, mass spectrometry (MS) provides structural data (molecular weight and molecular formula) of the examined compounds. Around 1 mg/mL of the crude and purified compounds were prepared separately for chemical profiling and molecular mass determination by LC-MS using a Dionex 3000 HPLC pump/Orbitrap mass spectrometer (Thermo Fisher Scientific, Bremen, Germany). A reverse-phase 5 µm C18 column (4.6 × 150 mm) (Hypersil, Thermo) was used and eluted using a gradient at a flow rate of 0.3 mL/min with 0.1% *v*/*v* formic acid in water and 0.1% *v*/*v* formic acid in acetonitrile as the two solvents (A and B) making up the mobile phase, as shown in Table 4. The ESI interface in negative ionization permitted the identification of [M-H]^−^. The spray voltages for the capillary and the cone were, respectively, −4.0 kV and 35 V. The flow rates of the sheath gas and auxiliary gas were, respectively, 50 and 15 arbitrary units. The ion transfer capillary had a temperature of 275 °C, and *m*/*z* 100–1500 provided the full scan data. The sample data were acquired and processed with the Xcalibur software (Thermo Fisher Corporation, Hemel Hempstead, UK).

#### 4.4.3. Evaluation of Antitumor Property: Cell Viability Assay

RPMI 1640 medium was used for the culturing and subculturing of human leukemia cell lines (U937). The medium was supplemented with penicillin and streptomycin (1% *v*/*v*), L-glutamine (1% *v*/*v*), and FCS (5% *v*/*v*), and U937 cells were cultured in desirable conditions, viz. a temperature of −37 °C, 100% humidity, and 5% CO_2_. To perform the cell viability assay, 100 µL of U937 cell suspension (containing 1 × 10^5^ cells/mL) was plated in each well and incubated for 24 h. Postincubation, cells were treated with crude and purified propolis samples prepared in varying concentrations (1.56–200 μg/mL) and incubated further for 24 h. DMSO was added and served as a positive control (to kill the cells completely); the negative control was the cells with medium. Following incubation, the resazurin indicator (10% Alamar blue) was loaded into the wells and incubated for an additional 24 h. The fluorescence of the plate was read using a Wallac Victor 2 microplate reader (λEx/EM: 560/590 nm), and cell viability was calculated and expressed as mean inhibitory concentration (IC50) values.

#### 4.4.4. Antitrypanosomal Assay In Vitro

An Alamar blue assay was carried out to evaluate the antitrypanosomal activity using a method described previously [37]. Principally, the test is based on viable cells metabolizing the blue resazurin dye to resorufin (a pink and fluorescent product). To perform the test, *Trypanosoma brucei* S427 cells at a seeding density of 2 × 10^5^ cells and propolis samples (20 mg/mL in 100% DMSO) that were double-diluted to varying concentrations (0.19 to 200 µg/mL) were prepared using Hirumi’s Modified Iscove’s medium 9 (HMI-9) as a diluent. Then, 100 μL of each propolis sample was added to a 96-well plate, followed by the addition of a trypanosome suspension (100 μL) to each well and incubation for 48 h at 37 °C in 5% CO_2_. After incubation, resazurin dye was added and incubated for a further 24 h under the same conditions. Following incubation, the fluorescence was recorded at λEx/EM: 544/620 nm using an FLUO star Optima (BMG Labtech, Offenburg, Germany).

#### 4.4.5. Statistical Analysis

The anticancer and antitrypanosomal activities were expressed as means ± standard errors. A paired *t*-test analysis was performed to determine the significance in the mean values of the anticancer and antitrypanosomal activities between the crude and isolated compounds. *p* < 0.05 was considered statistically significant.

## 5. Conclusions

The propolis extracts produced by the stingless bee *T. biroi* exhibited antiparasitic activity against the protozoan parasite *Trypanosoma* and anticancer activity against human myeloid leukemia cells. Based on the observed results, it can be confirmed that the bioactivity of propolis could be attributed to the presence of two major active compounds: 27-hydroxymangiferolic acid and 27-hydroxyisomangiferolic acid.

## Figures and Tables

**Table 1:** The most abundant components in the Philippine propolis fraction (Ph-2) when analyzed by reversed-phase LC-MS in negative ion mode.

Peak No.	Retention Time (min)	[M-1]	Chemical Formula	Delta (ppm)	Intensity
1	26.5	263.13	C_15_H_19_O_4_	1.473	E 6
2	39.57	405.27	C_24_H_37_O_5_	2.178	E 7
3	41.85	471.35	C_30_H_47_O_4_	2.284	E 7
4	49.64	469.33	C_30_H_45_O_4_	1.954	E 6
5	50.35	467.32	C_30_H_43_O_4_	2.155	E 7
6	53.72	455.35	C_30_H_47_O_3_	2.924	E 5

**Table 2:** Antineoplastic properties of the Philippine propolis sample and its fractions on U937 cells.

Sample Code	IC50 Mean Value (µg/mL)	Standard Deviation	% Relative Standard Deviation	*p* Value
Ph-2 fraction	129.6	17.6	13.6	<0.0001
Isomangiferolic acid	172.6	17.2	10.0	<0.0001
27-hydoxymangiferonic acid	164.7	37.0	22.5	<0.0001
27-hydroxyisomangiferolic acid	153.3	12.2	8.0	0.002
Pentamidine (µM)	13.3	1.0	7.6	<0.0001
Diminazen (µM)	29.6	2.2	7.3	<0.0001

**Table 3:** Antitrypanosomal activities of the Philippine propolis sample tested against *T. brucei S427*.

Sample Code	Mean (µg/mL)	Standard Deviation	% Relative Standard Deviation	*p* Value
Philippine crude	22.0	1.71	7.78	<0.0001
Ph-2 fraction	11.6	0.99	8.59	<0.0001
Isomangiferolic acid	21.4	1.83	8.58	<0.0001
27-hydoxymangiferonic acid	11.4	1.07	9.46	<0.0001
27-hydroxyisomangiferolic acid	13.9	1.20	8.68	<0.0001
Pentamidine (µM)	0.0045	0.0004	7.9334	<0.0001
Diminazen (µM)	0.0374	0.0017	4.4221	<0.0001

**Table 4:** Mobile phase system used in mass spectroscopy.

No.	Time (min)	A (%) Aqueous Phase (0.1% *v*/*v* Formic Acid in Water)	B (%) Organic Phase (0.1% *v*/*v* Formic Acid in Acetonitrile)	Flow (mL/min)
1	0	75	25	0.3
2	10	50	50	0.3
3	20	50	50	0.3
4	35	20	80	0.3
5	45	20	80	0.3
6	46	0	100	0.3
7	60	0	100	0.3
8	61	75	25	0.3
9	70	75	25	0.3

## Data Availability

Supplementary data available on request from the authors.

## Sample Availability

Samples of the compounds are not available from authors.
